# Bilinear Interpolation of Three–Dimensional Gain–Scheduled Autopilots

**DOI:** 10.3390/s24010013

**Published:** 2023-12-19

**Authors:** Sung Mo Koo, Timothy Sands

**Affiliations:** 1 Sibley School of Mechanical and Aerospace Engineering, Cornell University, Ithaca, NY 14853, USA; 2 Department of Mechanical Engineering (SCPD), Stanford University, Stanford, CA 94305, USA; dr.timsands@alumni.stanford.edu

**Keywords:** gain-scheduled autopilots, nonlinear control, bilinear interpolation, control and guidance, control gains, three-dimensional lookup table (3D-LUT)

## Abstract

Gain-scheduled autopilots have emerged as a dominant strategy to achieve adaptive control of coupled, non-linear engineering complexities, owing to an ability to adapt to changing operational conditions and uncertainties. This study focuses on utilizing bilinear interpolation of gain-scheduled autopilots, emphasizing enhanced system performance and robustness. Through a comprehensive investigation and comparative analysis using three disparate cases, advantages over conventional methods are revealed. Strengths and weaknesses of both simple and specialized variants (such as linear, and real-time gain-scheduling) are introduced. Three missile guidance case–studies utilize simulation time and miss distance figures of merit. Comparing the performance of bilinear interpolation and automatic instantiations to index–search, over comparable traveled distances, missile miss distances were improved 179% and 196% respectively with slightly improved computational burden.

## 1. Introduction

Gain-scheduled autopilots hold immense significance in the field of modern missile technology, as they address critical challenges and elevate the capabilities of guidance control in missile systems. As the complexity of military operations intensifies, the demand for highly adaptable countermeasures and precise missile guidance systems have become paramount. By providing dynamic control gains that cater to varying flight conditions, gain-scheduled autopilots offer a robust solution for missile defense. This study delves into the fundamental principles, working mechanisms, and functional advantages of various designs of gain-scheduled autopilots for missiles, highlighting the effectiveness of bilinear interpolation in control systems.

**Figure 1:**
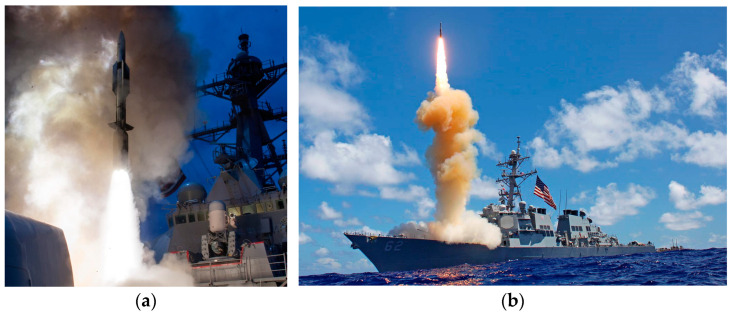
(a) Original RIM-174 Standard Extended Range Active Missile (ERAM), also known as Standard Missile-6 (SM-6) launched off the Hawaiian coast 6–13 April 2017 [1]. (**b**) guided-missile destroyer USS Fitzgerald (DDG 62) launches a Standard Missile-3 (SM-3) [2]. Images credit: U.S. Navy in accordance with image use policy [3]. Department of Defense photographs and imagery, unless otherwise noted, are in the public domain.

As a missile travels over vast distances (Figure 1), it encounters diverse atmospheric conditions that impact its aircraft stability, experiencing variations in altitude, velocity, and aerodynamic forces. Traditional fixed-gain autopilots suffer from limitations in adapting to the constantly changing environment and unexpected disturbances, leading to reduced accuracy and compromised performance. Accordingly, fixed-gain systems severely lack the ability to counter agile adversaries. Unmanned aerial systems (UAS), for instance, can exhibit unpredictable and erratic flight patterns, making them challenging targets for traditional fixed-gain autopilots.

Gain-scheduled autopilots, on the other hand, provide a dynamic and responsive solution. A gain-scheduled autopilot can tailor the control gains to suit each phase of the flight based on the real-time feedback and operational parameters. This adaptability significantly enhances the missile’s accuracy and maneuverability, increasing the chances of successful target engagement against evasive threats like UAS. Furthermore, in a mission-critical scenario where multiple missiles are employed in a salvo attack, gain-scheduled autopilots offer a crucial advantage. With the ability to dynamically adjust to surroundings, the missiles can adapt to the specific requirements of each individual missile, ensuring that the entire salvo operates cohesively. The ability to coordinate and synchronize multiple missiles makes gain-scheduled autopilots indispensable tools in modern warfare scenarios. By intelligently blending discrete control gains through simple algorithms, bilinear interpolation unlocks the full potential of gain-scheduled autopilots, contributing to the overall efficiency and reliability of missile guidance. The study explores the advantages of bilinear interpolation of control gains in a three-dimensional lookup table for gain-scheduled autopilots, including improved robustness, reduced control effort, and enhanced adaptability to handle complex, non-linear systems. The difference in this study is the seminal presentation of results conducted at this level of study. Presentation includes two-dimensional gain surfaces visualized in three-dimensional lookup table leading to comparison results based on simulation time (representing computational burden), range traveled, maximal normal acceleration, and miss distance.

### 1.1. Review of the Literature

Current long-range missile defense systems are seemingly much less effective than believed and suffer from severe limitations [4], particularly in light of recent improvements in decoys [5], where a long list of intercept failures was published in 2021 [6], where mitigation by sensor improvements was proposed the following year [7] in a proposal to utilize space–based sensors which was reinforced by the U.S. Air Force Association the same year in [8]. 

### 1.2. State of the Art Benchmarks

The following list highlights the current state of the art developments for high–fidelity six degree of freedom simulation:

1. In 2020, reference [9] illustrated ubiquitous use of simplified models and probabilistic assessments leading to recommendations on the number of interceptors necessary using a shoot-look-shoot mode of operations.
2. Russian President Putin boasted to have developed missiles traveling at twenty times the speed of sound [10] necessitating increased confidence in engagement analysis.
3. Tracy, et al. forwarded the notion that “Misperceptions of hypersonic weapon performance have arisen from social processes by which the organizations developing these weapons construct erroneous technical facts favoring continued investment”, and recommended rigorous, quantitative analysis [11].
4. In late 2022, The U.S. Army Combat Capabilities Development Command issued a technical report [12] elaborating benchmark automated gain–scheduling approach for three–loop autopilots for high–speed projectiles with supersonic flight envelopes.

These key benchmarks from the years 2020–2022 amplify the novelty of this study, namely proposal of bilinear interpolation in direct comparison to the current state of the art in comparisons where all features are held consistent, while only the autopilot technique is iterated revealing decision–quality advice for procurement (for example). Using a very recent published benchmark from the U.S. Army rationalizes selection for this study of the Standard Missile–6. 

### 1.3. Novelties Presented

The following proposals are developed in subsequent pages of this manuscript:

1. Automatic gain scheduling is proposed and compared to index–search as the comparative benchmark, where control gains are tailored to suit each phase of the flight based on the real-time feedback and operational parameters.
2. Bilinear interpolation is proposed and compared to index–search as the comparative benchmark by intelligently blending discrete controls. Linearized models are trimmed for each flight condition and control gains are tuned accordingly and stored at lattice points on partition of linear spaces of angle of attack, velocity, and gain. Actual flight conditions are located amidst the nearest lattice points and bilinear interpolation (using angle of attack and velocity) yields new control gain estimates.

## 2. Materials and Methods

### 2.1. Specification and Initialization

**Figure 2:**
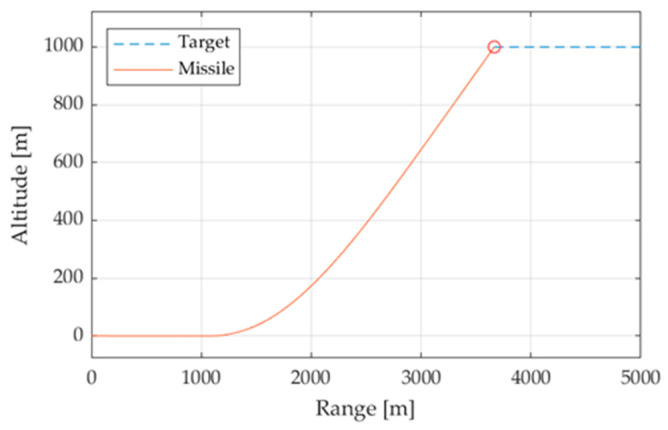
Flight simulation of missile and target. The red solid line represents the trajectory of the missile. The blue dotted line represents the trajectory of the target, cruising horizontally. The red circle indicates the point of impact.

The missile for the simulation is fictional, a replica of the original RIM-174 Standard Extended Range Active Missile (ERAM), also known as Standard Missile-6 (SM-6). SM is the most reliable type of surface-to-air missile, still favored by the United States Navy [13]. This study employs the aerodynamic fundamentals organized by Raytheon Missile Systems [14,15], and thus the most accurate simulation was expected by using SM, whose primary manufacturer is Raytheon. Possibly due to security purpose, some specifications of SM are not available publicly. While “…stabilization dynamics have a very wide bandwidth, in excess of 100 rad/sec…” [16], assumed control bandwidth is conservatively to be merely three Hertz. The parameters not listed in the table, like total thrust of the actuators, are instead computed from reference velocity (~Mach 5) using the drag equation. The final specification of the missile is shown in Table 1:

The study is focused on the relative performance of different designs of autopilots by comparing the simulation time and the miss distance. For this purpose, the target object does not require a complex, realistic geometry and it is simplified to a point mass with fixed flight coefficients. In contrast, the missile has varying flight coefficients for a more effective analysis of its aerodynamics. The initial position of the missile is defined as the origin. The missile initially travels parallel to the ground at a velocity of 1000 m/s. To make the iteration process more efficient, the target parameters are initialized relative to those of the missile. The target is placed 5000 m ahead and 1000 m above the missile’s initial position, traveling at a speed half of the initial speed of the missile, in the direction parallel to the surface and towards the missile, as seen in Figure 2.

### 2.2. Airframe Dynamics: Force and Moment

First, the environmental condition is established. The study utilizes the International Standard Atmosphere (ISA) model for altitudes between 0 to 20 km. The model takes in the current altitude of the missile and returns the climatic data elements, such as temperature, speed of sound, air pressure and air density, which are used to compute the full aerodynamics in the equations of motion.

To enhance the accuracy of the simulation, the force and moment coefficients of the missile are adjusted in accordance with the current flight condition. The coefficients are stored in a linearly spaced, three-dimensional partition of angle of attack, Mach number, and flight coefficients (α,M,C). The coefficients are parametrized as function of incidence angle and Mach number, represented as lattice points on the partition. When a flight condition is newly introduced on the grid as a nonlattice point, the simulation computes the relative position of the nonlattice point to the nearest lattice points. The new coefficient is estimated by a three-dimensional lookup table (‘3D-LUT’) using bilinear interpolation. 3D-LUT is explained with further detail in [17].

There are two major types of forces acting on the missile: axial force and normal force. The main axial force is drag acting on the missile body. The axial force coefficient is assumed to be constant, equal to that of a bullet with a spherical cap [18]. The study assumes the non–zero drag caused by the fins is negligible. The assumption is valid for fins with relatively small effective surface area relative to the missile’s frontal area (a mere 0.8 percent). Since the drag Equations (10)–(12) reveal linear scaling with area, drag fin may be safely approximated to be less than one percent of the missile drag. Table 2 defines terminology and is placed close to Equations (1)–(3) for convenience of the readership.

(1) $$C_{x_{f}}≅0$$

(2) $$C_{x_{\alpha }}=0.295$$

(3) $$C_{x_{t}}{=C}_{x_{\alpha }}+C_{x_{f}}$$

The lift force acts in the direction normal to the direction in which the missile travels. The largest contributors to the lift force are the main wings (or angle of attack) and the missile fins. The coefficient of the lift force created by the missile fins is estimated in [14]. The lift coefficient of the main wings is estimated using the non-linear equation (α,δ) introduced by Shamma, et al. [19]. Fin deflection is computed using the angle of attack and the velocity at the instant, allowing the simulation to compute the actual lift force. The total lift coefficient is the sum of the two lift coefficients.

(4) $$C_{z_{\alpha }}=0.000103\alpha^{3}-0.00945\alpha \left|\alpha \right|-0.170\alpha -0.034\delta$$

(5) $$C_{z_{f}}=1.2713$$

(6) $$C_{z_{t}}=C_{z_{\alpha }}+C_{z_{f}}$$

The pitch moment coefficient of the main wings is calcualted in Equation (7) [19]. The pitch moment coefficient due to fin deflection is provided in [14]. The total pitch moment coefficient is the sum of the moment coefficients.

(7) $$C_{m_{\alpha }}=0.000215\alpha^{3}-0.0195\alpha \left|\alpha \right|-0.051\alpha -0.206\delta$$

(8) $$C_{m_{f}}=7.5368$$

(9) $$C_{m_{t}}=C_{m_{\alpha }}+C_{m_{f}}$$

The resulting axial and normal forces are shown in Equations (10) and (11) where ρ and S are the air density and the reference area, respectively. Since the exact dimension of the missile elements is not published, the forces are assumed to share the same reference area, provided by Mracek, et al. [14]. The equation for the moment includes the moment arm l, equal to the reference length. The resulting forces and the moment are used to compute the flight metrics, such as position, velocity, acceleration, and attitude of the aircraft, in 3 degrees-of-freedom equations of motion. The metrics are fed into the autopilot, which determines the behavior of the aircraft. Table 3 defines terminology and is placed close to Equations (4)–(12) for convenience of the readership.

(10) $$F_{x}=\frac{1}{2}\rho V^{2}{C_{x}}_{t}S$$

(11) $$F_{z}=\frac{1}{2}\rho V^{2}{C_{z}}_{t}S$$

(12) $$M_{q}=\frac{1}{2}\rho V^{2}{C_{m}}_{t}Sl$$

Up to this point in the manuscript, ubiquitous (not novel), necessary topics have been introduced: Specification and simulation initialization, and flight dynamics (forces and moments). Before elaborating the three compared autopilots, one final ubiquitous topic is presented: the proportional navigation guidance law. 

### 2.3. Proportional Navigation Guidance Law

The study utilizes proportional navigation guidance (‘PN guidance’) for the entire homing phase [20]. PN guidance is one of the simpler guidance laws to implement. It only requires rate of change of line-of-sight (LOS) and closing velocity, allowing the missile to have the minimal sensory technology onboard [20]. The relative ease of implementation, however, does not signify its lack of performance. In fact, PN guidance has proven to be the most robust guidance system assuming a no-lag missile: a system that reacts instantly and exactly as commanded [20]. The conventional PN guidance law is shown in Equation (13).

(13) $$a_{m_{c}}=NV_{c}\dot{\lambda }$$

${a_{m}}_{c}$ is the commanded missile acceleration normal to the LOS. $V_{c}$ and $\dot{\lambda }$ are the closing velocity and the rate of change of LOS, respectively. $V_{c}$ is equal to $-\dot{R}$, where R is the range between the missile and the target. Note that $\dot{R}$ is a negative value during pursuit. For the system to be stable, the navigational gain N must be larger than 2.

### 2.4. Basic Fixed Gain Three-Loop Autopilot

**Figure 3:**
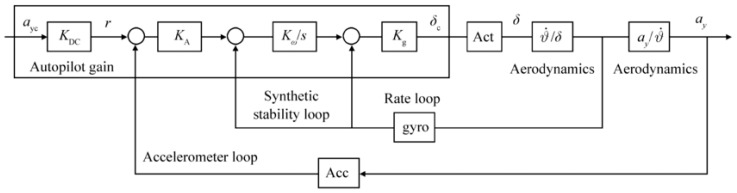
Topology of basic three-loop autopilot with fixed control gains [21].

The most basic form of autopilot is fixed gain autopilot (Figure 3). In fixed gain autopilot, the control gains are unchanged throughout the operation. The strength of the fixed gain autopilot is the simplicity of its structure. It neglects the change in the flight conditions and thus does not require the additional adjustments to optimize the gains during the operation. The key to the design is finding the gains that yield the desired time constant, ideally less than 0.2 s, and small steady-state error (~3%) prior to the main simulation.

The modelling of fixed gain autopilot requires the following steps:

1. Derive the nonlinear missile dynamics.
2. Derive the state representation of the linearized dynamics.
3. Tune the control gains for the nominal flight condition.

**Figure 4:**
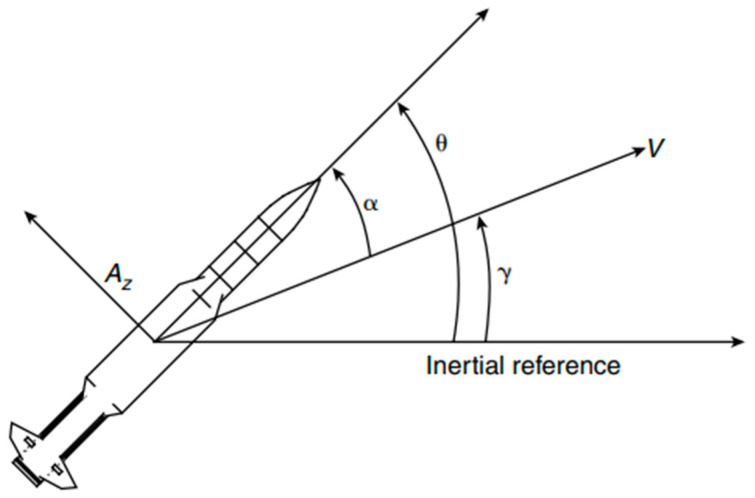
Nomenclature of critical angles in aerodynamics [9]. V is velocity vector; $A_{z}$ is normal acceleration acting on the missile body; α is angle of attack; θ is pitch angle, γ is flight-path-angle.

Nonlinear missile dynamics is derived from the longitudinal motion of the missile in the pitch plane. Specifically, the derivation of the nonlinear dynamics involves computation of the three critical angles in aerodynamics: the angle of attack, the flight-path-angle, and the pitch angle. The geometry of the angles is shown in Figure 4, the anatomy of a missile in longitudinal motion.

1. The velocity of the missile and the required normal acceleration are denoted $V_{m}$ and $A_{z}$, respectively. α is the angle of attack which describes the orientation of the missile relative to the airflow. γ is the flight-path-angle which describes the attitude of the missile. θ is the pitch angle which describes the orientation of the missile relative to the inertial reference frame. Naturally, the angle of attack is the difference between the pitch angle and the flight-path angle.
  (14) $$\alpha =\theta -\gamma \to \dot{\alpha }=\dot{\theta }-\dot{\gamma }$$
2. The rate of change in the flight-path-angle, $\dot{\gamma }$, can be expressed as a function of the vertical component of the normal acceleration, relative to the longitudinal axis, and the velocity vector. When the angle of attack is sufficiently small, the vertical component of the normal acceleration is assumed to be equal to the total acceleration. For maximum accuracy of the results, the estimation is not made.
  (15) $$\dot{\gamma }=\frac{A_{z}\cos\left(\alpha \right)}{V}$$
3. According to Newton’s 2nd law, the vertical acceleration of the missile is the vertical force applied to the missile divided by its mass. The final expression for $\dot{\gamma }$ is given in Equation (16). The nonlinear dynamics are shown in Equations (17) and (18), where Q is the dynamic pressure, d is the reference diameter, and $I_{YY}$, J is the moment of inertia [14,19,20].
  (16) $$\dot{\gamma }=\frac{F_{z}\cos\left(\alpha \right)}{mV}$$
  (17) $$\dot{\alpha }=\dot{\theta }-\frac{F_{z}\cos\left(\alpha \right)}{mV}$$
  (18) $$\ddot{\theta }=\frac{C_{m_{t}}QSd}{I_{YY}}\to \frac{M_{q}}{J}$$
4. With short-period approximation, the speed of the missile is assumed to be constant. Due to linear systems theory, nonlinear differential equations can be approximated in a linear form. $\ddot{\theta }$ is originally a function of α and δ. The analytical state-space model solved in the time domain is:
  (19) $$\left[\begin{array}{c} \dot{\alpha }\\ \ddot{\theta } \end{array}\right]=\left[\begin{array}{cc} -\frac{\partial F_{z}}{\partial \alpha }\frac{1}{mV} & 1\\ -\frac{\partial M_{q}}{\partial \alpha }\frac{1}{J} & 0 \end{array}\right]\left[\begin{array}{c} \alpha \\ \dot{\theta } \end{array}\right]+\left[\begin{array}{c} -\frac{\partial F_{z}}{\partial \delta }\frac{1}{mV}\\ -\frac{\partial M_{q}}{\partial \delta }\frac{1}{J} \end{array}\right]\delta$$

The reader is now free to consider utilization of transfer functions for treating the nonlinear system by converting the state space form in Equation (19) into a linear, time–invariant transfer function form. Table 4 defines terminology and is placed close to Equations (14)–(19) for convenience of the readership.

5. The state-space model in Equation (19) can be linearized via Taylor series expansion around the selected flight condition and only keeping the first-order terms. The final state-space model [14] may then be expressed in standard, linear state space form, Equation (20). With state, output and control definitions in Equation (21), the articulated standard state space equation becomes Equations (22)–(24).
  (20) $$\dot{x}=Ax+Bu$$
  (21) $$x=\left[\begin{array}{c} \alpha \\ q \end{array}\right];y=\left[\begin{array}{c} A_{z}\\ q_{m} \end{array}\right];\;u=\delta_{p}$$
  (22) $$\left[\begin{array}{c} \dot{\alpha }\\ \dot{q} \end{array}\right]=\left[\begin{array}{cc} \frac{1}{V_{0}}\left[\frac{\bar{Q}SC_{z_{\alpha }}}{m}-A_{x}\right] & 1\\ \frac{\bar{Q}SdC_{m_{\alpha }}}{I_{YY}} & 0 \end{array}\right]\left[\begin{array}{c} \alpha \\ q \end{array}\right]+\left[\begin{array}{c} \frac{\bar{Q}SC_{z_{f}}}{mV_{0}}\\ \frac{\bar{Q}SdC_{m_{f}}}{I_{YY}} \end{array}\right]\left[\delta_{p}\right]$$
  (23) y=Cx+Du
  (24) $$\left[\begin{array}{c} A_{z_{m}}\\ q_{m} \end{array}\right]=\left[\begin{array}{cc} \frac{\bar{Q}SC_{z_{\alpha }}}{mg}-\frac{\bar{Q}SdC_{m_{\alpha }}\bar{x}}{gI_{YY}} & 0\\ 0 & 1 \end{array}\right]\left[\begin{array}{c} \alpha \\ q \end{array}\right]+\left[\begin{array}{c} \frac{\bar{Q}SC_{z_{f}}}{mg}-\frac{\bar{Q}SdC_{m_{f}}\bar{x}}{gI_{YY}}\\ 0 \end{array}\right]\left[\delta_{p}\right]$$
6. Raytheon provides a fully linearized model for both stable and unstable systems [14], well aligned with the purpose of the study. The linearized model for an unstable system is:
  (25) $$\left[\begin{array}{c} \dot{\alpha }\\ \dot{q} \end{array}\right]=\left[\begin{array}{cc} -1.064 & 1\\ 290.26 & 0 \end{array}\right]\left[\begin{array}{c} \alpha \\ q \end{array}\right]+\left[\begin{array}{c} -0.25\\ -331.40 \end{array}\right]\left[\delta_{p}\right]$$
  (26) $$\left[\begin{array}{c} A_{z_{m}}\\ q_{m} \end{array}\right]=\left[\begin{array}{cc} -123.34 & 0\\ 0 & 1 \end{array}\right]\left[\begin{array}{c} \alpha \\ q \end{array}\right]+\left[\begin{array}{c} -13.51\\ 0 \end{array}\right]\left[\delta_{p}\right]$$
7. Refer to Equations (A1)–(A4) in Appendix A for the stable system solution [14]. The corresponding open loop transfer functions for the actuator model are:
  (27) $$\frac{A_{z_{m}}}{\delta_{p}}=\frac{-13.51s^{2}+16.29s+44,800}{s^{2}+1.064s-290.26}$$
  (28) $$\frac{q_{m}}{\delta_{p}}=\frac{-331.4s-424.7}{s^{2}+1.06s-290.28}$$
8. For simplicity of the study, the actuator model is often assumed to be a linear system due to its structural complexity and nonlinear behavior. However, unlike the popular belief, the discrepancy raised from the assumption is not significant enough to hurt the validity of a simulation [22]. Hence, the second-order approximation of the actuator model is:
  (29) $$\frac{\delta \left(s\right)}{\delta_{c}(s)}=\frac{\omega_{a}^{2}}{s^{2}+2\zeta_{a}\omega_{a}s+\omega_{a}^{2}}$$
  (30) $$\frac{\delta \left(s\right)}{\delta_{c}(s)}=\frac{22,500}{s^{2}+210s+22,500}$$

Table 5 defines terminology and is placed close to Equations (20)–(30) for convenience of the readership.

9. Using the linearized dynamics and the simplified actuator model, the control gains are tuned to yield the time constant less than 0.2 s. The fixed autopilot gains for the nominal condition are:
  $$K_{DC}=0.0187;\;K_{A}=0.9188;K_{\omega }=0.0168;K_{g}=0.6832$$

Equations (20)–(30) may be input to the simulation whose topology is depicted in Figure 3 permitting formulation of output–to–input relationships (e.g., transfer functions) that are tuned to solve for gain values to meet performance specifications. Higher calculated values of gains lead to faster responding autopilots, where the overall autopilot gain is labeled $K_{DC}$ and the missile acceleration gain is labeled $K_{a}$. The rate loop (including integral rate) is control by gains $K_{g}$ and $K_{\omega }$ respectively. 

### 2.5. Various Gain-Scheduled Autopilots

Gain-scheduled autopilot is an augmented version of fixed gain autopilot with the ability to improve flight performance in the middle of operation. Unlike fixed gain autopilots which have predetermined, unchanging control gains, gain-scheduled autopilot swaps out the gain values from an index of previously stored gains, allowing the autopilot to perform well in the current flight condition. Particularly, the ability to adjust gain values is most highlighted at the end game of pursuit where the flight conditions are most rapidly changing.

Due to its effectiveness, gain-scheduled autopilot is widely adopted in modern systems. It is still being actively studied and thus has many variations in its design.

#### 2.5.1. Index-Search

Index-search is the simplest form of gain-scheduled autopilot. The study considers a three-loop autopilot with four control gains: $K_{DC}$, $K_{a}$, $K_{\omega }$, and $K_{g}$. The control gains are tuned at each flight condition and stored in their respective indices. When an aircraft reaches a certain flight condition, the gain values are selected strictly from the stored gain values. The number of sets of gain values is equal to the number of available flight conditions. The design process is as following:

1. Trim and linearize the nonlinear plant models for each flight condition.
2. Tune the control gains for each linear mode.
3. Store the linear controllers into a family of controllers.
4. Swap the control gains based on the current flight condition.

The trimming and linearization process is like the process previously introduced in Section 2.4. In short, one must find steady-state values of elevator deflection and pitch rate that yield steady angle of attack at a chosen velocity. The angle of attack and the velocity are called scheduling variables, used to determine the actual flight condition. Once a family of linear controllers is established, the autopilot can swap the control gains. As the scheduling variables shift during the operation, autopilot chooses the gains from one of the pre-existing flight conditions that is in the closest vicinity to the actual flight condition. Although the method ensures a more desirable performance than a fixed-gain autopilot, it is unable to fully describe the plant behavior of controllers that are not linearized in prior and hence not included in the family.

#### 2.5.2. Bilinear Interpolation in 3D-LUT

Bilinear interpolation allows the autopilot to estimate control gains for plant models that are unlisted in the family. Unlike index-search, the number of sets of gains is not limited to the number of linear controllers. Rather, the autopilot can generate control gains as necessary regardless of the current flight condition. Hence, the transition of control gains is much smoother in the second method than the first method.

**Figure 5:**
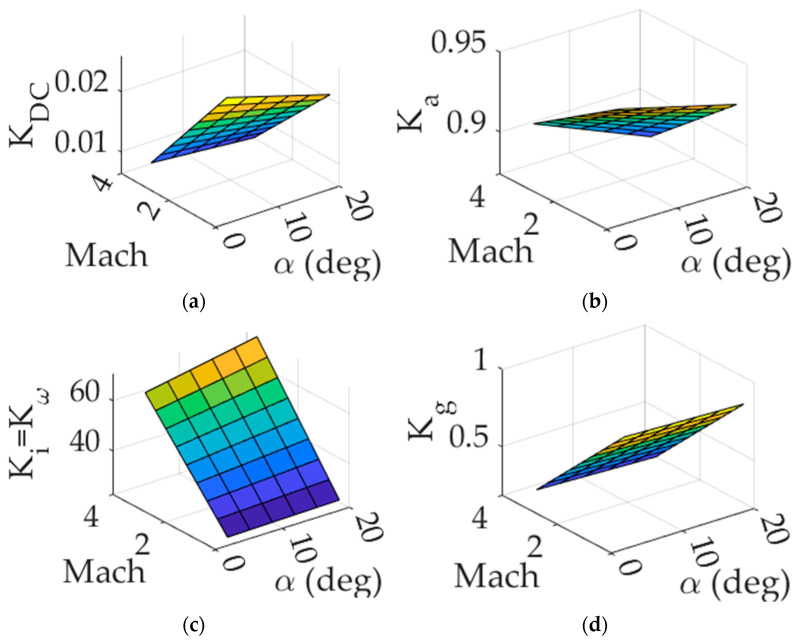
Two-dimensional gain surfaces visualized in three-dimensional lookup table. (**a**) $K_{DC}$, (**b**) $K_{a}$, (**c**) $K_{\omega }$, (**d**) $K_{g}$.

1. Trim and linearize the nonlinear plant models for each flight condition.
2. Tune the control gains for each linear model.
3. Create a 3D-LUT, a linearly spaced partition of α, V, and K (depicted in Figure 5);
4. Store the tuned gains to their respective lattice points (α, V) on the partition.
5. When aircraft enters a new flight condition, determine the relative position of the new nonlattice point to the nearest lattice points.
6. Perform bilinear interpolation (α, V) to estimate the new control gain.

The center of the design is the estimation of the control gains via bilinear interpolation. Consider a three-dimensional lookup table (‘3D-LUT’), a partition with control gains as its lattice points whose coordinates are (α, V). The autopilot determines where the new condition, represented as a non–lattice point, falls on the partition when an aircraft enters a new flight condition. Particularly, autopilot checks the position of the new point by comparing α and V to those of the nearest points. Using the difference in the positions of the non–lattice point and the nearest points, the new control gains are computed by performing two consecutive bilinear interpolation. However, it must be noted that the generated control gains may not work well with nonlinear plant models [23]. Reference [24] illustrated how controls derived for linearized plants generally do not satisfy optimization criteria for the nonlinear system and offered amelioration using Pontryagin’s systems theories. Accordingly, such analysis is recommended for future research. Just last year, Banginwar [25] offered an initial proposal of such applications (still neglecting nonlinear coupling terms from the transport theorem), where follow–on efforts should incorporate the nonlinear coupling terms. Alternatively, a trajectory tracking control approach for an uncertain surface vessel using the new cascade structure of adaptive reinforcement learning algorithm and kinematic controller, feed-forward term was offered in [26], while an adaptive reinforcement learning optimal tracking control algorithm was presented in [27,28] for an underactuated surface vessel subject to modeling uncertainties and time-varying external disturbances.

#### 2.5.3. Automatic Real-Time Tuning

The third method is to parametrize the control gains as first-order polynomials of the scheduling variables. A conventional method of tuning control gains involves a significant number of variables. This novel approach, the parametrization of gains, reduces the number of variables to four, allowing the system to compute the gains instantaneously with minimal computational burden. Consider a gain parametrized as a polynomial function of the scheduling variables [25].

(31) $$K\left(\alpha ,V\right)=K_{0}+K_{1}\alpha +K_{2}V+K_{3}\alpha V$$

The simplest way to tune the polynomial coefficients is to convert the polynomials into tunable surfaces in MATLAB. The tunable surfaces can be tuned automatically using MATLAB functions, such as <*systune*> and <*looptune*>. The control gains must be initialized prior to the tuning. The tuning requirements, time constant and steady-state error, are equal to those introduced in Section 2.4. The detailed tutorial on the automatic tuning of tunable surfaces is included in [25].

The obvious strength of the method is that the tuned control gains are always best for their respective flight conditions. Unlike the previous methods, the gains are neither compromised nor estimated from linear models, allowing the autopilot to perform at utmost accuracy in nonlinear environments. However, automatic tuning requires the most computational power which raises the system requirements on board.

## 3. Results

The relative performance of the three methods of gain-scheduled autopilot is investigated. Each method is tested in three missile behavioral profiles. Particularly, the missile is configured to (1) stable-low-velocity profile, (2) stable-high-velocity profile, and (3) unstable-high-velocity profile. It is expected that an autopilot with a more advanced method for obtaining the control gains will deliver finer results. Respectively, a shorter simulation time and a smaller miss distance signify greater efficiency and higher accuracy.

### 3.1. Simulation Results

#### 3.1.1. Simulation Time

The simulation time, or the time taken for target acquisition, varies noticeably across the autopilots. As shown in Table 6, the third autopilot exhibits the shortest simulation time while the first autopilot requires a significantly longer time on average (~5%). The second autopilot reaches the target almost simultaneously with the third autopilot, running nearly equally in efficiency. Initially, the control effort was expected to heavily affect the simulation time. Rather, it appears the simulation time is dictated by how effectively a controller commands a missile to pursue the best trajectory. For instance, the third autopilot has the most fluid transition in its control gains, allowing the controller to give fin demands that prevent the missile from escaping the trajectory.

#### 3.1.2. Range Traveled

To help visualize the actual efficiency of each autopilot, the total range traveled by the target is collected, as shown in Table 7. On the exterior, the longer the range covered by the target the more imminent the threat becomes. The difference between the range covered by the targets in the second and the third autopilots is almost negligible. Meanwhile, the first autopilot (index–search) needed approximately 2% longer distance to intercept the target.

#### 3.1.3. Miss Distance

The most important factor in determining the effectiveness of an autopilot is the miss distance. The study assumes that a missile must denotate near its target within 10 m for effective blast fragmentation. In other words, the miss distance must be smaller than 10 m for a reliable hit-to-kill. As shown in Table 8, the miss distance of the first autopilot in the third case is frighteningly close to the threshold distance. In other words, the first autopilot may not be reliable in scenarios with more evasive targets. The miss distance of the second and the third autopilot is safely within the explosion radius. Particularly, both the second and third autopilots exhibit minimal miss distance, less than 1m, in all of the cases provided.

In summary, the second autopilot, which uses bilinear interpolation, demonstrates capability comparable to the third autopilot in efficiency and accuracy. The simple interpolation algorithm reduces the control effort significantly while maintaining nearly perfect guidance for interception. Owing to its simple structure, the second autopilot can be easily fitted to any system and be a compelling candidate for control engineers seeking a robust, accessible alternative to the conventional autopilots.

### 3.2. Validation of Results

**Figure 6:**
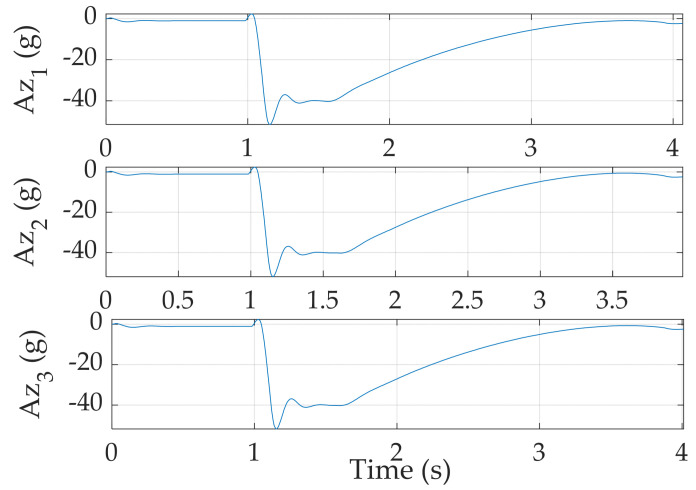
Acceleration acting normal to the surface of the missile. The underscore numbers represent their respective case numbers.

A particular innovation presented is computational experiments validating relative performances of the three disparate autopilots in direct comparison. The comparison illustrates a key impact of this research. One final comparison is consideration of structural integrity necessitating examination of acceleration forces. It is crucial to examine whether the second autopilot ensures the structural integrity of the missile while pursuing a target. Most structural failures occur when the acceleration experienced by the missile is too large or when the rate of change in the fin angle is too drastic. In particular, the acceleration in the direction normal to the surface of the missile and the maximum fin demand are investigated and depicted in Figure 6.

**Figure 7:**
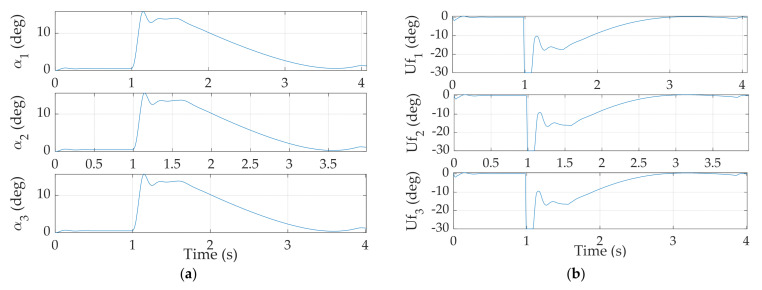
(**a**) Angle of attack; (**b**) Commanded fin deflection. Any discontinuity signifies the selected autopilot method may not be applicable to a real system.

The normal acceleration must be lower than 60 G, the typical maximum acceleration experienced by an aerobatic missile [29]. As shown in the Table 9, the missile is most likely to suffer the largest acceleration in Case 2 and Case 3 where the missile maintains high velocity. Their respective maximum acceleration is 52.055 G and 53.392 G. All of the maximum accelerations are below the threshold with reasonable margin, implicating the feasibility of the selected autopilot. Moreover, angle of attack and fin deflection do not exhibit any cusp or discontinuity in their trends, as seen in Figure 7, meaning the autopilot is viable for a real mission.

## 4. Discussion

Bilinear interpolation of control gains in 3D-LUT provides a simple and robust solution to control design of gain-scheduled autopilot. The straightforward interpolation algorithm makes the autopilot easy to implement, thus reducing control effort and complexity of the technology onboard. In fact, the overall performance of the autopilot with bilinear interpolation is comparable to that of the state-of-the-art autopilot with severely higher computational burden. In conclusion, the study shows that bilinear interpolation of control gains offers a practical and competent way to enhance control system performance, adapt to changing conditions, and achieve robustness and stability, making it a valuable tool in the arsenal for aerospace control. Engineers and researchers can leverage the insights gained from this study to design highly efficient and robust autonomous control systems for a wide range of applications not limited to aerial guidance, accelerating advancements in adaptive control technology, and fostering the development of more sophisticated and reliable autonomous systems.

### 4.1. Multivariate Performance Comparison

A multivariate performance comparison of the three autopilots studied is included in Table 10.

### 4.2. Recommended Future Research

Future research should explore innovative ways of leveraging big data and machine learning algorithms to learn and adapt to system dynamics in real-time. By incorporating data-driven gain-scheduling, gain-scheduled autopilots may better address unforeseen disturbances and changing system characteristics, ultimately leading to more robust and resilient autonomous control systems. Furthermore, nonlinear optimization [23] seemingly holds promise for future improvements. 

## Figures and Tables

**Table 1:** Specifications of the missile for the simulation.

Description	Value	Unit
Total Missile Mass	160	kg
Initial Missile Velocity	1000	m/s
Maximum Axial Acceleration	400	$m/s^{2}$
Reference Area	0.050	$m^{2}$
Reference Length	0.300	m
Pitch Moment of Inertia	180	kg$m^{2}$
Actuator Bandwidth	3.000	rad/s
Maximum Fin Deflection	±30	deg

**Table 2:** Table of proximal variables and nomenclature ^1^.

Variable/Acronym	Definition	Variable/Acronym	Definition
$C_{x_{f}}$	Drag coefficient of fins	$C_{z_{\alpha }}$	Lift coefficient of wings
$C_{x_{\alpha }}$	Drag coefficient of body	$C_{z_{f}}$	Lift coefficient of fins
$C_{x_{t}}$	Total drag coefficient	$C_{z_{t}}$	Total lift coefficient

^1^ Such tables are offered throughout the manuscript to aid readability.

**Table 3:** Table of proximal variables and nomenclature ^1^.

Variable/Acronym	Definition	Variable/Acronym	Definition
$F_{x}$	Axial force	$C_{m_{\alpha }}$	Moment coefficient of wings
$F_{z}$	Normal force	$C_{m_{f}}$	Moment coefficient due to fins
$M_{q}$	Pitch moment	$C_{m_{t}}$	Total moment coefficient

^1^ Such tables are offered throughout the manuscript to aid readability.

**Table 4:** Table of proximal variables and nomenclature ^1^.

Variable/Acronym	Definition	Variable/Acronym	Definition
$I_{YY}$	Moment of inertia	$\dot{\alpha }$	Rate of change in AOA
J	Analytic body inertia	$\dot{\gamma }$	Rate of change in FPA
m	Mass of missile	$\ddot{\theta }$	Pitch acceleration

^1^ Such tables are offered throughout the manuscript to aid readability.

**Table 5:** Table of proximal variables and nomenclature ^1^.

Variable/Acronym	Definition	Variable/Acronym	Definition
g	Gravitational acceleration	$\delta_{p}$	Fin deflection
$\omega_{a}$	Actuator frequency	$A_{z}$	Normal acceleration
$\zeta_{a}$	Actuator damping ratio	$A_{x}$	Axial acceleration

^1^ Such tables are offered throughout the manuscript to aid readability.

**Table 6:** Simulation time and percent comparison.

Case	Method	Simulation Time [seconds]	Percent Difference
1	Index-search	4.233	0%
	Bilinear interpolation	4.071	−3.90%
	Automatic	4.063	−4.09%
2	Index-search	4.192	0%
	Bilinear interpolation	3.980	−5.19%
	Automatic	3.974	−5.34%
3	Index-search	4.300	0%
	Bilinear interpolation	4.016	−6.83%
	Automatic	4.001	−7.20%

**Table 7:** Range traveled by target and percent comparison.

Case	Method	Range Traveled [meters]	Percent Difference
1	Index-search	1352.96	0%
	Bilinear Interpolation	1341.85	−0.82%
	Automatic	1340.88	−0.89%
2	Index-search	1339.31	0%
	Bilinear Interpolation	1310.77	−2.15%
	Automatic	1310.02	−2.21%
3	Index-search	1362.71	0%
	Bilinear Interpolation	1324.90	−2.81%
	Automatic	1322.03	−3.03%

**Table 8:** Miss distance and percent comparison.

Case	Method	Miss Distance [meters]	Percent Difference
1	Index-search	6.8411	0%
	Bilinear Interpolation	0.4684	−174.37%
	Automatic	0.0710	−195.89%
2	Index-search	8.1884	0%
	Bilinear Interpolation	0.4552	−178.94%
	Automatic	0.0702	−196.60%
3	Index-search	9.8919	0%
	Bilinear Interpolation	0.4583	−182.29%
	Automatic	0.0863	−196.54%

**Table 9:** Inspection of aerodynamic stress on the structure of the missile.

Case	Maximum Normal Acceleration [G]	Threshold Margin [G]
1	53.813	6.187
2	52.055	7.945
3	53.392	6.608

**Table 10:** Multivariate performance comparison of three autopilot options: Index–search, bilinear interpolation, and automatic gain scheduling.

Case	Method	Miss Distance [meters] Percent Difference	Range Traveled [meters] Percent Difference	Simulation Time Percent Difference
1	Index-search	0%	0%	0%
	Bilinear Interpolation	−174.37%	−0.82%	−3.90%
	Automatic	−195.89%	−0.89%	−4.09%
2	Index-search	0%	0%	0%
	Bilinear Interpolation	−178.94%	−2.15%	−5.19%
	Automatic	−196.60%	−2.21%	−5.34%
3	Index-search	0%	0%	0%
	Bilinear Interpolation	−182.29%	−2.81%	−6.83%
	Automatic	−196.54%	−3.03%	−7.20%

## Data Availability

Data supporting reported results can be obtained by contacting the corresponding author.

## Appendix A

Raytheon provides the solution to the short period dynamics of a lightly damped, fast, stable system [2]. The state space representation of a stable system is

(A1) $$\left[\begin{array}{c} \dot{\alpha }\\ \dot{q} \end{array}\right]=\left[\begin{array}{cc} -1.064 & 1\\ -290.26 & 0 \end{array}\right]\left[\begin{array}{c} \alpha \\ q \end{array}\right]+\left[\begin{array}{c} -0.25\\ -331.39 \end{array}\right]\left[\delta_{p}\right]$$

(A2) $$\left[\begin{array}{c} A_{z_{m}}\\ q_{m} \end{array}\right]=\left[\begin{array}{cc} -101.71 & 0\\ 0 & 1 \end{array}\right]\left[\begin{array}{c} \alpha \\ q \end{array}\right]+\left[\begin{array}{c} -13.51\\ 0 \end{array}\right]\left[\delta_{p}\right]$$

and the corresponding open loop transfer functions are as follows:

(A3) $$\frac{A_{z_{m}}}{\delta_{p}}=\frac{-13.51s^{2}+10.91s+29,780}{s^{2}+1.064s+290.26}$$

(A4) $$\frac{q_{m}}{\delta_{p}}=\frac{-331.4s-280.3}{s^{2}+1.064s+290.26}$$
